# Validation of Housekeeping Genes to Study Human Gingival Stem Cells and Their *In Vitro* Osteogenic Differentiation Using Real-Time RT-qPCR

**DOI:** 10.1155/2016/6261490

**Published:** 2015-12-30

**Authors:** Ihsène Taïhi, Ali Nassif, Tsouria Berbar, Juliane Isaac, Ariane Berdal, Bruno Gogly, Benjamin Philippe Fournier

**Affiliations:** ^1^Laboratory of Molecular Oral Physiopathology, INSERM UMRS 1138, Cordeliers Research Center, 75006 Paris, France; ^2^Paris-Descartes, Pierre and Marie Curie, and Paris-Diderot Universities, UFR Odontology, 75006 Paris, France; ^3^AP-HP, Hospital Complex Henri-Mondor Albert-Chenevier, CIC-BT-504, 94000 Creteil, France; ^4^Reference Center for Dental Rare Disease, Rothschild Hospital, 75012 Paris, France

## Abstract

Gingival stem cells (GSCs) are recently isolated multipotent cells. Their osteogenic capacity has been validated *in vitro* and may be transferred to human cell therapy for maxillary large bone defects, as they share a neural crest cell origin with jaw bone cells. RT-qPCR is a widely used technique to study gene expression and may help us to follow osteoblast differentiation of GSCs. For accurate results, the choice of reliable housekeeping genes (HKGs) is crucial. The aim of this study was to select the most reliable HKGs for GSCs study and their osteogenic differentiation (dGSCs). The analysis was performed with ten selected HKGs using four algorithms: Δ*Ct comparative method, GeNorm, BestKeeper*, and *NormFinder*. This study demonstrated that three HKGs, *SDHA, ACTB*, and *B2M*, were the most stable to study GSC, whereas *TBP, SDHA*, and *ALAS1* were the most reliable to study dGSCs. The comparison to stem cells of mesenchymal origin (ASCs) showed that *SDHA/HPRT1* were the most appropriate for ASCs study. The choice of suitable HKGs for GSCs is important as it gave access to an accurate analysis of osteogenic differentiation. It will allow further study of this interesting stem cells source for future human therapy.

## 1. Introduction

Gingival stem cells (GSCs) are emerging new tools that have been recently isolated from human gingival tissue [1], a tissue with high healing capacity [2]. These cells can differentiate into osteoblasts, chondrocytes, and adipocytes when appropriately induced [1, 3]. An easy, noninvasive, and scar-free access to gingival tissue makes GSCs interesting cells and a potential source for future regenerative medicine applications, in order to replace or repair diseased tissues and organs [2].

The osteogenic differentiation of GSCs has been evaluated* in vitro* and confirmed using qualitative approaches such as Alizarin Red S staining [1]. This potential may be used to treat large maxillary bone defects. GSCs may be an alternative to stem cells derived from mesoderm, because they share their embryonic origin with facial bones, that is, cephalic neural crests [4, 5]. In order to better characterize these cells and their* in vitro* osteogenic differentiation, molecular biology quantitative assays can be of high interest, using gene expression study. This approach is more accurate than histochemical qualitative methods to study GSCs and confirm their stemness and differentiation potencies. To achieve this purpose, reverse transcription quantitative Polymerase Chain Reaction (RT-qPCR) technique is considered the most accurate and reliable method, owing to its sensitivity, real-time detection of reaction progress, and the amplification of very small quantities of mRNA [6].

However, reproducibility problems and biases have been recently discussed [7]. Indeed, many factors may affect the RT-qPCR accuracy. These are related to operator's manipulation: pipetting, treating samples, cell culture, the amount of starting material, different conditions of harvesting cell samples, RNA extraction yield, mRNA quality, and homogeneity taken from tissues or cells. DNase treatment, reverse transcription enzyme specificity, the efficiency of reverse transcription reaction, the type of fluorescence, and the temperatures of qPCR cycles may also provoke differences [6].

In order to minimize errors related to these factors, data normalization is mandatory. Other solutions have been recently proposed, such as using robots, RNA extraction kits, or normalized kits from cell culture to cDNA synthesis, which showed high sensitivity, accuracy, and reproducibility [8]. However, these techniques remain expensive and not accessible to all research centers. The standardized normalization of qPCR results against reference genes thus remains the most common method to have reproducible and reliable results [6].

Reference genes or housekeeping genes (HKGs) are internal reaction control [9]. It is considered that these HKGs reflect basic metabolism implicated in the general processes and mechanisms of cell cycle and hence are supposed not to be affected or regulated by experimental conditions. Several criteria are required for a reliable reference gene: the expression level must be unchanged by experimental conditions, and its variability must be as minimal as possible in the different samples. It would be preferable that the cycle threshold (Ct) of such a HKG is close to the one of the gene of interest. It is often recommended to use at least two HKGs or more, because only one may lead to biases or may be regulated by experimental conditions without the possibility to control it [6]. Thus, HKG choice is a crucial step of the RT-qPCR protocols, and the investigator must carefully validate it. However, such information remains often lacking [10].

To avoid mischoices, several statistical approaches and algorithms have been proposed [11–14]. These methods have been employed on different mesenchymal stem cells, but to our knowledge not yet on oral stem cells [15]. The objective of this study is to choose the most reliable HKGs to study GSCs and their osteoblast differentiation by RT-qPCR. To validate these HKGs, we will assess the 10 most used HKGs with four published algorithms:* the *Δ*Ct comparative method*,* GeNorm*,* BestKeeper*, and* NormFinder*.

## 2. Materials and Methods

The study has been carried out thanks to the collaboration between the Laboratory of Molecular Oral Physiopathology, INSERM UMRS 1138, Cordeliers Research Center, Paris, France, and the Hospital Complex Henri-Mondor Albert-Chenevier, CIC-BT-504, Creteil, France.

Written informed consents were obtained from patients in accordance with Helsinki Declaration Principles, taking into account the ethical, legal, and regulatory norms and standards for research in France (Loi Huriet number 91-73), as well as the applicable international norms and standards.

Our research protocol was submitted to the research ethics committee: Centre d'Investigations Cliniques de Creteil-Henri Mondor (Biothérapie) CIC-BT-504, directed by Pr. José COHEN, which specifically approved this study.

### 2.1. Isolation and Growth of Stem Cells: Gingival Stem Cells (GSCs)

GSC cultures were obtained from six healthy donors (*n* = 6), after their informed consent. Gingival tissues were collected from gingiva of tooth extraction sites from the donors during surgery. Stem cells were isolated either by explants technique, followed by fibroblast colony forming units (CFU-F) assay, or by enzymatic digestion with Collagenase II (Sigma-Aldrich) [16–18]. Eight GSCs samples were finally obtained (six samples from enzymatic digestion technique and two samples from explants technique). They were seeded in 35 mm Petri dishes, proliferated with 10 mL of Dulbecco's Modified Eagle Medium Low Glucose (DMEM-LG), GlutaMAX, and pyruvate Supplement (Gibco-Life Technologies, Carlsbad, CA, USA). The medium was supplemented with 10% of foetal calf serum (FCS; Gibco), 10 mL/L Penicillin-Streptomycin (5 UI/mL, Gibco), 1% of nonessential amino acids (NEAA; Gibco), and 2.5 mg/L of Amphotericin B (250 *μ*g/mL; Gibco). Culture medium was filtered in 0.22 *μ*m pore size filters before use. Cell cultures were maintained at 37°C in a humidified 5% CO_2_ incubator. Culture media were changed twice a week, with supplementation of L-Ascorbic Acid 2-Phosphate (50 *μ*g/mL; Sigma-Aldrich), until they attempted 70 to 80% of confluence. Cells were then harvested after Trypsin-EDTA treatment and centrifugation. They were frozen at −80°C and stored until use for RNA extraction. GSCs were characterized using flow cytometry with stem cells markers: CD29, CD73, CD90, CD105, CD45, and HLA-DR [1].

### 2.2. Adipose Derived Stem Cells (ASCs; Purchased from ZenBio Company, USA)

Six samples of ASCs (*n* = 6), mesenchymal stem cells of adipose tissue origin, used as control, were also cultured in the same conditions, in early passages (1–5), in order to compare them to GSC. When reaching 70–80% of confluence, they were collected and frozen at −80°C for RNA extraction.

### 2.3. Osteoinduction

GSC (*n* = 8) and ASC (*n* = 6) were cultured in 6-well plates. When attempting 70 to 80% of confluence, osteogenic differentiation was induced by DMEM-LG supplemented with 20% of FCS, 10 mL/L of Penicillin-Streptomycin (5 UI/mL), 1% of NEAA, 2.5 mg/L of Amphotericin B (250 *μ*g/mL), adding to 50 *μ*g/mL of L-Ascorbic Acid 2-Phosphate, 100 nM of dexamethasone, and 10 mM of *β*-glycerophosphate [19]. Culture media were changed every 72 hours, by adding L-Ascorbic Acid 2-Phosphate. Dexamethasone was supplemented every 7 days. After 21 days, differentiated GSC (dGSC) and ASC were either harvested after Trypsin-EDTA treatment and centrifugation, frozen at −80°C for RNA extraction, or fixed in PFA 4%/Sucrose 5% and conserved at 4°C for staining.

### 2.4. Histochemical Staining

**Figure 1:**
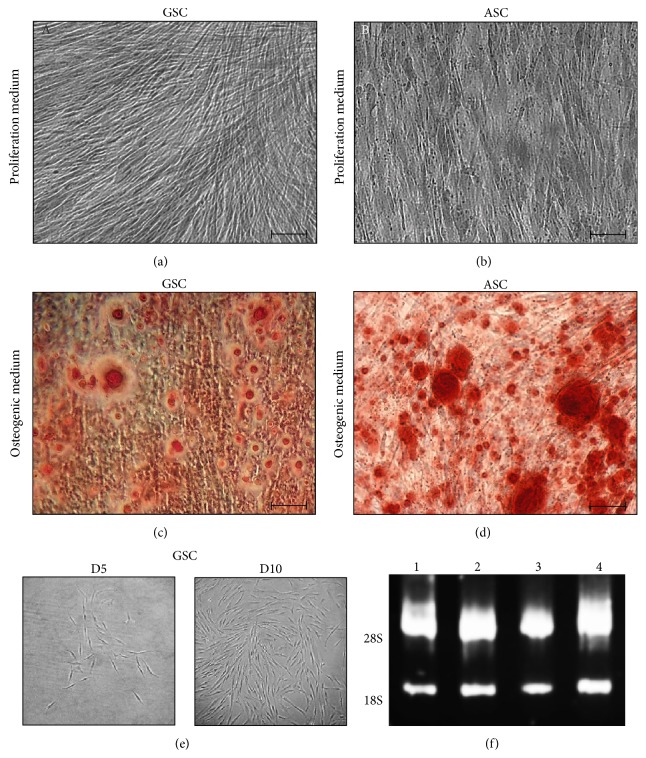
(a), (b), (c), and (d) Alizarin Red S Staining of GSC and ASC after 21 days of osteoblast induction. (a) and (b) GSC and ASC were cultured in proliferation medium. No differentiation is noticed. (c) and (d) GSC and ASC were cultured in osteogenic medium. Calcium mineral deposits confirmed osteoblast differentiation in both stem cells after 21 days of culture. Bar scale = 100 *μ*m. (e) Colony forming units for GSCs. Limiting dilution of GSCs shows their ability to form colonies after 5 days of culture. (f) Agarose gel electrophoresis: total RNA quality. All RNA samples showed absence of degradation and a high degree of integrity. Upper bands: 28S, lower bands: 18S, and lanes 1 to 4: random RNA samples from GSC and ASC.

To confirm osteoinduction, fixed cells were washed with phosphate-buffered saline (PBS, Gibco), followed by two distilled water rinses and incubation in fresh Alizarin Red S solution (1 g/100 mL in distilled water, Sigma-Aldrich), pH around 4.10, for 30 min. The wells were then rinsed repeatedly with distilled water, and calcium deposits were observed (Figure 1).

### 2.5. Total RNA Extraction and cDNA Synthesis

Total RNA was isolated (GSC *n* = 8, ASC *n* = 6, and differentiated GSC *n* = 8), using ReliaPrep RNA Cell Miniprep System kit (Promega), according to manufacturer's instructions. This kit fits cell culture and incorporates a recombinant DNase treatment step. RNA concentrations and purity were assessed by a NanoDrop spectrophotometer (Thermo Scientific, Wilmington, DE, USA). The purity was determined by measuring the absorbance at 260 nm/280 nm. The ratio *A*_260_/*A*_280_ is expected to be between 1.8 and 2.0. RNA quality was also confirmed by agarose gel electrophoresis, choosing 4 random samples, which confirmed the absence of ribosomal RNA degradation, with a 28S/18S ratio around 2 (Figure 1(f)).

2 *μ*g of RNA of each sample was reverse-transcribed using SuperScript II Reverse Transcriptase (Invitrogen) initiated by random primers and oligo-dT primers, as described in the provided protocol. The final volume was 20 *μ*L. The latter was 20-fold diluted (v/v) to have the same final concentration of 5 ng/*μ*L of cDNA. Reactions were also prepared without the reverse transcriptase enzyme, which gave minus RT products to confirm the absence of genomic DNA contamination in qPCR reactions.

### 2.6. Selection of Reference Genes

Primer sequences for the 10 candidate housekeeping genes in the study of human stem cells,* TBP, SDHA, HPRT1, GAPDH, ALAS1, ACTIN, RPS18, RPII, UBC,* and* B2M*, were obtained from NCBI primer-blast tool, USA (Table 1). They were selected with an amplicon size <250 bp, spanning an exon–exon junction when possible, with no polymorphism, and the ideal melting temperature *T*_*m*_ was 60°C (with a maximum difference of 3°C between every two primers). For primer nucleotides, they should end with a C or G residue, and CG content was ranged between 50 and 60%. The cycle threshold (Ct) was ranged from 15 to 30. The same tool and settings were used to generate the primers for the analysis of stem cells osteogenic differentiation, such as* RUNX2.*

### 2.7. RT q-PCR Assay

The assay was carried out in triplicate in a 96-well format in the Bio-Rad CFX96 Real Time System (Bio-Rad Laboratories, USA). The reaction volume was 15 *μ*L. For each sample, the real-time PCR mixtures consisted of 3 *μ*L cDNA (≈15 ng of cDNA) and 12 *μ*L of mixture of 7.5 *μ*L of SYBR Green Supermix (Bio-Rad), 0.45 *μ*L of primer (250 nM final of each sense and antisense sequence of the primer), and 4.05 *μ*L of RNase/DNase-free sterile water. The assay included negative controls (nuclease-free water) and controls with minus RT to detect reagent contamination and the presence of genomic DNA, respectively. In order to define the efficiency (*E*) of each primer, serial dilutions (five dilutions: from half to five times alternately) of one GSC sample which expresses the genes of interest and the housekeeping genes and relative standard curves were generated. Efficiency *E* was calculated as follows: PCR efficiency = (10^[−1/slope]^ − 1) × 100.

The reactions were run for 35 cycles following this thermal cycling profile: (1) 95°C for 3 min, (2) denaturation at 95°C for 5 sec, (3) primer annealing step at 60°C for 20 sec (the most optimal temperature for all primer pairs after performing preliminary real-time RT q-PCR assays).

To confirm primer specificity, a melting curve analysis was performed after each amplification, ranging from 65°C to 95°C, with temperature increasing steps of 0.5°C every 5 sec. In all negative samples, no fluorescent signal was detected, or at very late cycle (more than 10 cycles after the Ct). This ensured of the quality of RNA extraction in all the samples.

### 2.8. Analysis of Gene Expression

**Figure 2:**
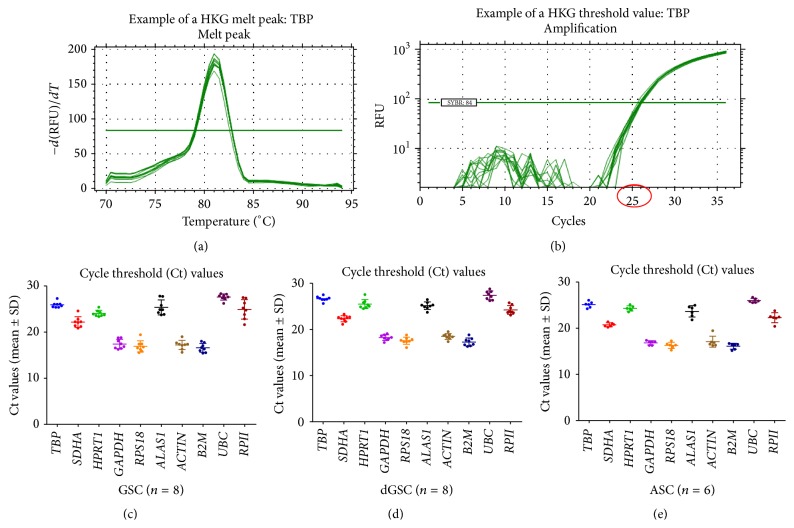
PCR reaction values and expression levels of the 10 candidate reference genes. (a) Melt peak temperature was determined for each reference gene to confirm the specificity of each primer. (b) Threshold values were manually set for each reference gene to calculate cycle threshold (Ct) values. (c), (d), and (e) Expression levels (*Ct values*) were determined for all reference genes throughout all the samples: (c) GSCs (*n* = 8), (d) dGSCs (*n* = 8), and (e) ASCs (*n* = 6). The central bars correspond to the mean Ct values; the upper and lower bars represent the standard deviation. These values were used in ΔCt and* BestKeeper* algorithms.

In order to assess the most stable reference genes to study GSC, ASC, and dGSC, Ct of all samples were calculated (Figures 2(c), 2(d), and 2(e)). Ct is defined as the number of cycles needed for the fluorescence signal to reach a specific threshold of detection and is therefore reversely correlated to the input amount of total RNA [20]. These values were used in Δ*Ct and BestKeeper* algorithms. Values of relative gene expression or normalized values (*R*) were also calculated for use in* GeNorm* and* NormFinder* analyses (see Supplementary Material 1 in Supplementary Material available online at http://dx.doi.org/10.1155/2016/6261490). For each HKG, Ct values were logarithmically transformed for all the samples; the lowest Ct was used as a calibrator against which change is given. For each sample, ΔCt (the subtraction of cycle threshold between the studied sample and the calibrator) is calculated. The investigators use the efficiency (*E*_HKG_) of each primer, and the formula is the following:

(1) The normalized valueR:  R=EHKGΔCtCalibrator sample−studied sample.

#### 2.8.1. The ΔCt Comparative Method

This approach is based on the comparison of Ct differences, or ΔCt values of each “pairs of genes” within each sample. The objective is to determine if the reference gene has an increased or decreased level of deviation in ΔCt among the samples when compared to the other reference genes. If the ΔCt remains stable when analyzed in different samples, it means that these two genes are stably expressed all among the samples or that they are coregulated [13]. ΔCt mean is calculated for each pair of genes and between every two groups of HKGs. All genes are taken into account and all possible “gene combinations” are compared. Then, the standard deviation values of each ΔCt set of each pair of genes is determined.

Reference genes are ranked according to the arithmetic mean of standard deviation values, which must be as low as possible to be the most stable. Box-and-whiskers charts can also be generated to show the distribution of ΔCt values in each pair of genes in the samples and allow us to compare ΔCt variability of each reference gene against all others. The bar is a line which represents the median (middle of dataset). The 75 and 25 percentiles represent the upper and lower limits of the boxes, and the whiskers refer to the highest and lowest ΔCt values among the samples excluding outsiders. The HKG with the lowest variability is the most stable [21].

#### 2.8.2. GeNorm Analysis

This method determines, among set of candidate genes, the two most stable ones that share a similar expression profile throughout all studied samples [22]. For each two candidate genes (gene *j* and gene *k*), using normalized values (*R*, here *a*), the algorithm calculates the array of log_2_ transformed expression ratios and the standard deviation *V* of the pairwise variation of this gene toward all other genes, as shown in the following formula:

(2) Ajklog2⁡a1ja1k,log2⁡a2ja2k,…,log2⁡amjamk=log2⁡aijaiki=1→m,Vjkst.devAjk,

where *A*_*jk*_ is array of pairwise variation of normalized values of* gene j* toward* gene k* for all samples; *V*_*jk*_ is standard deviation of *A*_*jk*_ values for* gene j* toward* gene k*.

Then the stability value (*M*), defined as the average or arithmetic mean of the standard deviations of this gene, is calculated [14]:

(3) $$\begin{array}{c} M_{j}=\frac{\sum_{k=1}^{n}V_{jk}}{n-1}. \end{array}$$

The algorithm ranks HKGs toward their *M* values, from the most stable with the lowest *M* to the least stable. If *M* value is more than 1.5, the gene is considered not stable and is removed from the analysis.* GeNorm* recalculates, hence, *M* values for the remaining stable genes and ranks reference genes from the most stable to the least stable one. Stepwise exclusion of the least stable gene with the highest *M* value will ultimately result in the two most stable genes that cannot be further ranked and selected a pair of genes as the most stable. The other genes are hence ranked based on their highest compatibility with *M* of the first pair [14].

In order to define accurately the number of HKGs needed for the normalization,* GeNorm* proposes a second step of calculation of the normalization factor, which is the variation between each pair of genes consecutively ranked from the most to the least stable. If the values are smaller than 0.15, there is no need to add a new HKG. An Excel Add-In method is available to make* GeNorm* analysis and graphs excel, http://medgen.ugent.be/~jvdesomp/genorm/.

#### 2.8.3. BestKeeper Analysis

Unlike Δ*Ct comparative method* and* GeNorm* software, BestKeeper considers not only “intergene” relation, but takes also into account the “intragene” variation, which assesses samples variation in the same gene. In this approach, the ideal HKGs are expected to have a stable expression in the same tissue or sample [12]. For this, standard deviation (SD: ±Ct) and covariance (CV: %Ct) are the two important values to assess HKG stability. For a studied reference gene, SD of Ct values must be as low as possible and must not be >1; otherwise it will be excluded. A second step consists of studying the correlation with* BestKeeper index* and selecting the most correlated reference gene to this index. The tool simulates an ideal HKG, or the “best” HKG, for which it calculates, for each sample, a* BestKeeper index* defined as the geometric mean of Ct values of all HKGs for this sample, as shown in the following formula:

(4) BestKeeper  Index=√zCP1×CP2×CP3×⋯×CPz;

see [23].

Then it compares this index to each reference gene by a pairwise correlation and regression analyses and calculates a coefficient of correlation (*r*) and the probability *p*. “*r*” should be as close to 1 as possible and “*p*” as low as possible. The* BestKeeper index* can also be compared to a further ten genes, for the same samples, to decide whether they are differentially expressed. Moreover, this algorithm calculates invariances, InVar (±Ct), of each sample to validate its stability; these values must be <3-fold the least one. A freely Excel based spreadsheet software exists and allows us to calculate automatically these values to make the choice of the most stable HKGs easier. It has been established that sample size has a minimal effect on this tool [23], http://www.gene-quantification.de/bestkeeper.html#download.

#### 2.8.4. NormFinder Analysis

This model takes into account the influence of variation of gene expression in the same sample and coregulation between different individuals [11]. For each candidate gene, the algorithm calculates a stability value *p*, taking into account the intra- and intergroup variances. The reference gene which has the smallest *p* value is ranked as the most stable, as it has the smallest variation over all samples. By creating subgroups, the algorithm can also select the most appropriate candidate genes to study two groups of samples, choosing the ones with the minimal intra- and intergroup variations. Intragroup variation must be as small as possible, and intergroup variation ≈ 0. The results are affected by the number of samples and are more accurate when *n* increases (*n* ≥ 8). It also requires at least 5 to 10 HKGs.

In order to compare the 4 algorithms and select the most stable HKGs, we applied RefFinder (http://www.leonxie.com/referencegene.php), a web-based comprehensive tool, which uses the currently available algorithms, Δ*Ct comparative methods [13]*,* GeNorm [14]*,* BestKeeper [12]*, and* NormFinder [11]*, and assigns an appropriate weight to an individual gene and calculates the geometric mean in order to rank all the studied reference genes.

## 3. Results

### 3.1. GSC Osteogenic Induction and RNA Quality Control

Eight GSC samples were obtained from healthy patients during surgical treatment. Six samples of ASCs were purchased and cultured in the same conditions. Both GSCs and ASCs were induced into osteogenic differentiation in duplicate for 21 days. Cellular matrix in proliferation medium was negative to the Alizarin Red S staining (Figures 1(a) and 1(b)). Mineral nodules and matrix were present in osteogenic medium and confirmed with this histochemical staining for both stem cells (Figures 1(c) and 1(d)). This stresses that GSCs are capable of osteogenic differentiation* in vitro* like ASCs, which are already known to form minerals* in vitro* under these conditions of culture. GSCs were CD29+, CD73+, CD90+, CD105+, CD45−, and HLA-DR− by flow cytometry analysis (data not shown) and form CFU-F (Figure 1(e)). RNA of both GSC and ASC was collected, with a yield of more than 1 *μ*g. The analyses of purity with the NanoDrop spectrophotometer showed suitable ratios *A*_260_/*A*_280_ with values around 2.00. RNA quality was confirmed randomly for 4 samples of GSCs and ASCs by agarose gel electrophoresis, insuring the integrity of ribosomal RNA, with two bands of 18S and 28S (Figure 1(f)).

### 3.2. The Choice of the Most Reliable HKGs for GSCs, dGSCs, and ASCs

In order to select the most appropriate and stable HKGs to study GSC and their osteogenic differentiation, expression of ten reference genes chosen according to previous published studies [15, 23, 24] was assessed. These reference genes were* TBP* (TATA-binding protein),* SDHA* (succinate dehydrogenase complex, subunit A, flavoprotein),* HPRT1* (hypoxanthine guanine phosphoribosyltransferase I),* GAPDH* (glyceraldehyde-3-phosphate dehydrogenase),* RPS18* (40S ribosomal protein S18),* ALAS1* (5-Aminolaevulinate synthase),* ACTB* (Beta-actin (*β*-actin)),* B2M* (Beta-2-microglobulin),* UBC* (Ubiquitin C), and* RPII* (50S ribosomal protein L9). Gene information are available and primer's efficiencies were between 82.5% and 103% (Table 1).

RT-qPCR were performed for 35 cycles in GSC samples (*n* = 8), GSCs after osteogenic induction (referred to as differentiated GSCs: dGSC) (*n* = 8), and compared to ASCs (*n* = 6). A melting curve analysis showed a single product at the expected melt temperature for each reference gene (Figure 2(a)). Results were analysed with a logarithmic scale and a threshold level was manually selected for each reference gene to detect the expression levels as cycle threshold (Ct) values for all samples (Figure 2(b)). Ct values were collected for each group of samples (Figures 2(c), 2(d), and 2(e)). In all samples,* GAPDH*,* RPS18*,* B2M*, and* ACTB* had Ct values below 20 cycles and hence had an early expression, unlike* SDHA*,* TBP*,* HPRT1*,* ALAS1*,* UBC*, and* RPII* that had lowest expression values. The normalized values (*R*): *R* = *E*_HKG_^ΔCt(Calibrator  sample−studied  sample)^ were calculated for each sample using Ct values (Supplementary Material 1). Data were treated with four algorithms:* the *Δ*Ct comparative method [13]* and* GeNorm [14]* for inter-gene relation,* BestKeeper [12]* and* NormFinder [11]* for intra- and intergene correlation (Table 2).

### 3.3. The ΔCt Comparative Method

**Figure 3:**
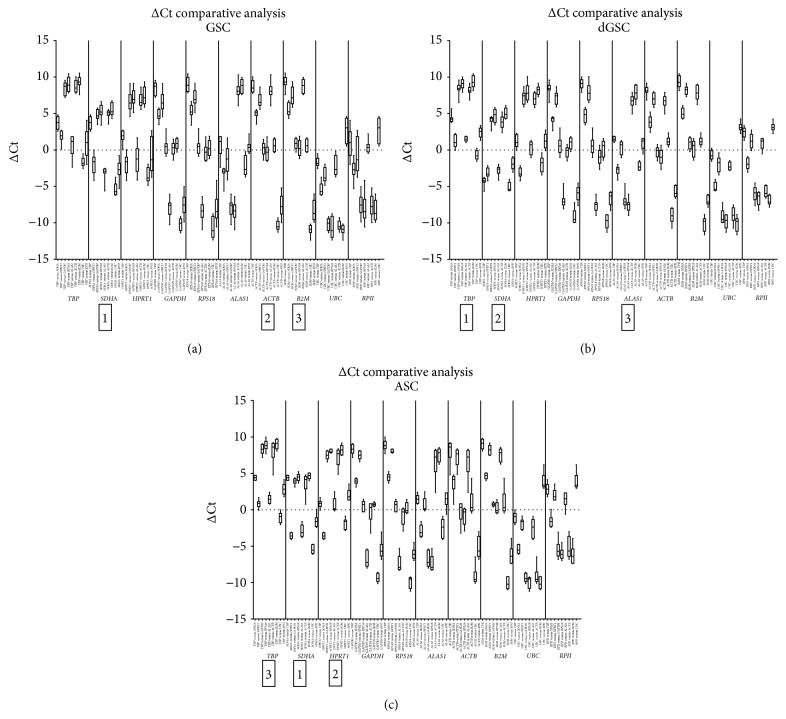
Ranking of 10 HKG in GSC (a), dGSC (b), and ASC (c) with ΔCt comparative method. ΔCt variability with pairwise comparisons of the complete set of candidate housekeeping genes shown as boxes and whiskers: medians (lines), 25th and 75th percentiles (Boxes). Genes with lowest variability were the most stable.

This method is based on comparing the variability of expression levels of each “pair of genes” within all the samples, in order to identify the reference gene with the lowest variability to be ranked as the most stable. The method was applied in each group of samples separately: GSCs, dGSCs, and ASCs. For each pair of genes, ΔCt values were calculated in all the samples, as well as the ∆Ct mean and the standard deviations (StdDevs) of ΔCt values (for GSCs, Table 3; for ASCs and dGSCs, Supplementary Material 2). The arithmetic mean of StdDevs was then identified for each reference gene and HKGs were ranked according to these values. This is shown in box-and-whiskers charts with all possibilities for all the 10 HKGs (Figures 3(a), 3(b), and 3(c)). In GSC samples,* SDHA*,* ACTB*, and* B2M* were the most stable genes (StdDev mean = 0.97, 0.98, and 0.98, resp.).* HPRT1* and* RPII* were the least stable and had a higher variability within the samples (StdDev mean = 1.38 and 1.64, resp.). In dGSCs,* TBP*,* SDHA*, and* ALAS1* showed the lowest variability in the samples as compared to the other genes, with a StdDev mean of 0.69, 0.72, and 0.78, respectively, whereas* GAPDH* and* RPS18* showed the highest variability. In ASCs, the most stable genes were* SDHA*,* HPRT1*, and* TBP*, with the lowest StdDev means (0.66, 0.71, and 0.77, resp.), while* ALAS1* and* ACTB* were the least stable ones.

Finally, the intergene analysis by ΔCt comparative method ended with a recommendation to normalize GSCs data using* SDHA*,* ACTB*, and* B2M*, dGSCs with* TBP*,* SDHA*, and* ALAS1*, and ASCs with* SDHA*,* HPRT1*, and* TBP*.

### 3.4. GeNorm Analysis

Normalized expression values of each group: GSCs, dGSCs, and ASCs were entered in the Excel spreadsheet under the format recommended by the authors, respectively [14].

**Figure 4:**
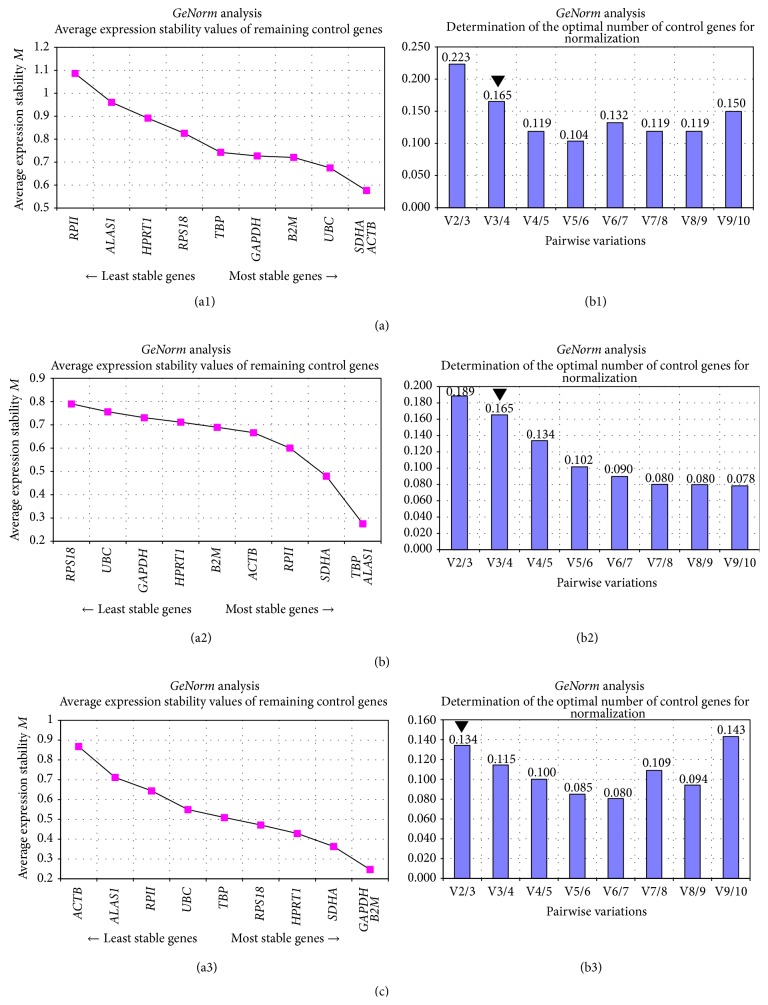
Gene expression stability and minimal number of genes needed in (a) GSC (*n* = 8), (b) dGSC (*n* = 8), and (c) ASC (*n* = 6), by GeNorm. ((a1), (a2), and (a3)) Average expression stability values (*M*) for GSC, dGSC, and ASC. A lower *M* value indicated a more stable expression. ((b1), (b2), and (b3)) Pairwise variation value below 0.16 with the least number of reference candidates used is considered optimal for GSC, dGSC, and ASC.

Firstly, the stability value *M* was calculated for all HKGs, and reference genes were ranked from the most stable, with the lowest *M* values, to the least stable ones, as shown in Table 4. Graphs were generated showing the average expression stability *M* values, selecting the most stable pair of genes, according to which it ranks the other genes (Figure 4(a)).

For GSCs (*n* = 8), the most stable genes were* SDHA*,* B2M*, and* ACTB*, (*M* = 0.892, 0.919, and 0.924, resp.).* RPII* was the least stable (*M* = 1.588) and excluded from analysis (*M* > 1.5). Hence, RPS 18 and* ALAS1* were the least stable (*M* = 1.162, 1.163, resp.).* SDHA* and* ACTB* were selected as the most stable pair of genes (Figure 4(a1)). Regarding dGSCs (*n* = 8),* TBP*,* SDHA*,* ALAS1*, and* RPII* were the most stable with *M* values, 0.656, 0.677, 0.729, and 0.771, respectively.* UBC* and* RPS18* had the highest *M* values (*M* = 0.858; 0.925) and were considered the least stable genes.* TBP* and* ALAS1* were thus selected as the best pair of genes (Figure 4(a2)). As for ASCs (*n* = 6),* SDHA*,* HPRT1*, and* TBP* were the most stable (*M* = 0.619, 0.656, and 0.717, resp.).* ALAS1* and* ACTB* were the least stable (*M* = 1,081; 1,491).* GAPDH* and* B2M* were the optimal pair of genes according to the software (Figure 4(a3)).

Pairwise variation analysis by calculating two sequential normalization factors (NF_*n*_ and NF_*n*+1_) suggested that the optimal number of reference genes to study teach group of stem cells was three HKGs for GSCs and dGSCs (*V*_3/4_ = 0.165 for both of them, which is around 0.15 as suggested by the software) (Figures 4(b1) and 4(b2)) and two reference genes for ASCs (*V*_2/3_ = 0.134 below 0.15) (Figure 4(b3)).

Finally, the intergene analysis by* GeNorm* recommended* SDHA*/*ACTB* and* UBC* to study GSCs,* TBP*/*ALAS1* and* SDHA* for dGSCs, and* GAPDH*/*B2M* or* SDHA/HPRT1* to study ASCs (Table 4).

### 3.5. BestKeeper Analysis

**Figure 5:**
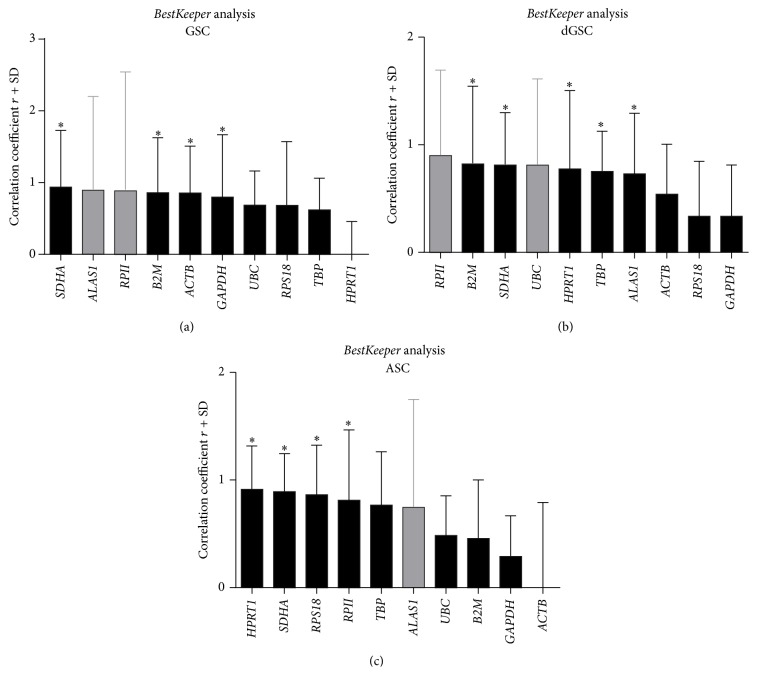
Ranking of the most stable HKGs with* BestKeeper* analysis for GSC (a), dGSC (b), and ASC (c). Bars represented the coefficient of correlation *r*, while error bars represented the standard deviation (SD). Ideal reference gene had low SD and a high *r*. ^*∗*^ *p* ≤ 0.05.

Raw Ct values of each group (GSCs (*n* = 8), dGSCs (*n* = 8), and ASCs (*n* = 6)) were uploaded to* BestKeeper* software in the form of an Excel spreadsheet. Firstly,* BestKeeper* excluded candidate HKGs with highest standard deviation (SD) and the covariance (CV). For GSCs,* RPII* and* ALAS1* had the highest SD (1.66,1.31) and CV (6.99,5.18), respectively, and were therefore excluded from analysis. Using pairwise correlation analysis and regression analysis,* BestKeeper* then compared the intergene relations of remaining candidate genes based on its index.* UBC*,* HPRT1*,* TBP*, and* RPS18* had a low correlation with* BestKeeper index* (*r* = 0.685, −0.050, 0.622, and 0.684, resp.) and thus were also excluded from analysis, even though they may have low SD and CV like* UBC*,* HPRT1*, and* TBP* (Figure 5(a)). Among the remaining genes,* SDHA* had the lowest variation (SD = 0.79, CV = 3.55) and the best correlation with* BestKeeper index* (*r* = 0.938, *p* < 0.001) followed by* B2M* (*r* = 0.861, *p* < 0.006),* ACTB* (*r* = 0.841, *p* < 0.007), and* GAPDH* (*r* = 0.799, *p* < 0.017) as summarized in Table 5(a).

For dGSCs (*n* = 8), all HKGs had low variances, with the least variability for* TBP* (SD = 0.37, CV = 1.40) and* ACTB* (SD = 0.46, CV = 2.48) and the highest variability for* RPII* (SD = 0.79, CV = 3.27) and* UBC* (SD = 0.80, CV = 2.92). Then the correlation with* BestKeeper index* excluded* GAPDH* (*r* = 0.335),* RPS18* (*r* = 0.336),* ACTB* (*r* = 0.540), and* ALAS1* (*r* = 0.731). Gene ranking from the most correlated was* B2M* (*r* = 0.823, *p* < 0.012),* SDHA* (*r* = 0.812, *p* < 0.014),* HPRT1* (*r* = 0.776, *p* < 0.024), and* TBP* (*r* = 0.752, *p* < 0.031).* RPII* and* UBC* had a high correlation with* BestKeeper index* (*r* = 0.900, *p* < 0.002 and *r* = 0.810, *p* < 0.015, resp.); however, they were the least stable, as shown formerly with the highest SD and CV values (Figure 5(b)). Consequently, the final ranking was* B2M*,* SDHA*,* HPRT1*, and* TBP* (Table 5(b)).

For ASCs (*n* = 6), ALAS 1 was first excluded (SD = 1.01, CV = 4.23). Then, the correlation with* BestKeeper index* showed a low *r* for* ACTB* (*r* = −0.142),* GAPDH* (*r* = 0.291),* B2M* (*r* = 0.458), and* UBC* (*r* = 0.486), which were also excluded (Figure 5(c)). The most correlated HKGs with* BestKeeper index* were* HPRT1* (*r* = 0.914; *p* < 0.011),* SDHA* (*r* = 0.893; *p* < 0.017),* RPS18* (*r* = 0.864; *p* < 0.027), and* RPII* (*r* = 0.813; *p* < 0.049) as shown in Table 5(c).

Based on these results, the most reliable HKGs with* BestKeeper* software were* SDHA*,* B2M*, and* ACTB* for GSCs,* B2M* and* SDHA* for dGSCs, and* HPRT1*,* SDHA*, and* RPS18* for ASCs.

### 3.6. NormFinder Analysis

Normalized values of the three groups were introduced into* NormFinder* software as recommended by the authors [11]. The algorithm is presented as a free complement in Excel program. The analysis calculates the value of stability *ρ* of each reference gene and ranks them from the most stable with the lowest *ρ* to the least stable.

For intragroup analysis,* NormFinder* ranked* SDHA*,* B2M*, and* ACTB* as the most stable (*ρ* = 0.248, 0.300, and 0.317, resp.) for GSCs.* TBP*,* SDHA*, and* ALAS1* were the most stable for dGSC (*ρ* = 0.222, 0.234, and 0.335, resp.), and* SDHA*,* TBP*, and* RPS18* for ASCs (*ρ* = 0.063, 0.224, and 0.246, resp.) (Table 6).

**Figure 6:**
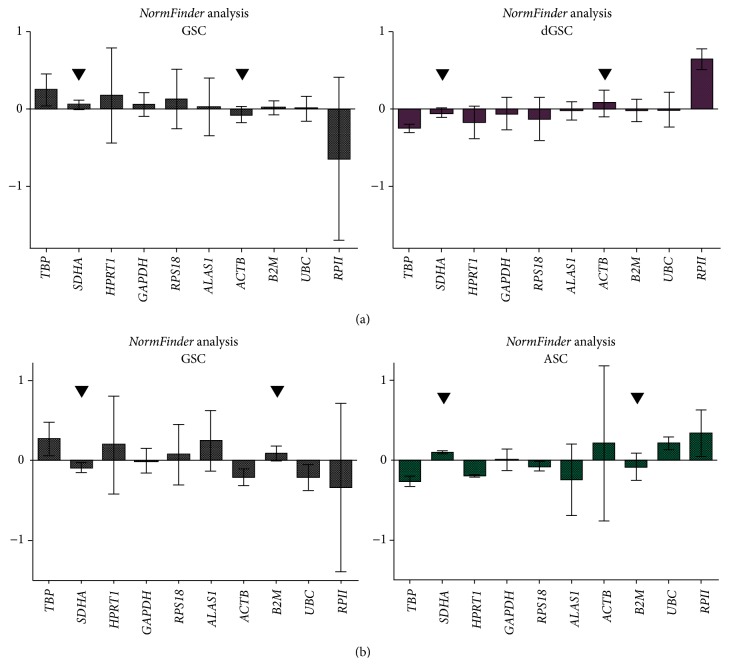
Determination of the most stable reference genes with* NormFinder* For GSC versus dGSC (a) and GSC versus ASC (b). Bars represent intergroup variances, while error bars represented the average of intragroup variances. Ideal reference gene had intergroup variation as close to zero as possible and error bars as small as possible.

Further analysis was realized in order to select the most appropriate reference genes to compare gene expression of GSCs to dGSCs and to ASCs. For this purpose, two pairs of subgroups were created: GSCs versus dGSCs and GSCs versus ASCs.* NormFinder* calculated intra- and intergroup variances, as shown in charts in which error bars presented intragroup variances and bars referred to intergroup variances (Figures 6(a) and 6(b)).

As for GSCs versus dGSCs, although* ALAS1* and* UBC* displayed low intergroup variances,* ALAS1* had a high intragroup variance in GSCs and* UBC* in dGSC, hence not suitable for the study (Figure 6(a)).* RPII* had the highest intergroup variance in GSCs and dGSCs and also the highest intragroup variance in GSCs; this gene was then ranked as the least stable one.* SDHA* and* ACTB* were selected by the algorithm as the most suitable to compare GSCs to dGSCs, with the lowest intergroup variances and intragroup variances close to zero.

Concerning GSCs versus ASCs,* GAPDH* and* RPS18* had low intervariances, but their intragroup variances were high:* GAPDH* in both GSCs and ASCs and* RPS18* in GSCs. Hence, they were not the most stable genes to study these subgroups.* RPII* had the highest intergroup variance and was the least stable gene.* SDHA* and* B2M* had the lowest intergroup variances and were also stable inside each group, with the lowest intragroup variances. Consequently, they were selected to compare gene expression between GSCs and ASCs.

Thus,* NormFinder* allowed us to confirm gene ranking of* GeNorm* for the three groups of samples, GSCs, dGSCs, and ASCs and, moreover, enabled us to select the most appropriate genes to compare GSCs versus dGSCs:* SDHA* and* ACTB*, and GSCs versus ASCs:* SDHA* and* B2M*.

### 3.7. Final Ranking of HKGs for GSCs, dGSCs, and ASCs

Based on the four algorithms, an additional control was performed with RefFinder, a tool that uses only Ct values and calculates the geometric mean of overall ordering of all reference genes and suggested a final ranking of the most stable housekeeping genes. Results are summarized in Table 7.

### 3.8. Effect of the Choice of Stable HKGs

In order to validate the importance of choosing the optimal reference genes to normalize RT-qPCR data, two analyses of gene expression of GSCs were performed based on the former results.

**Figure 7:**
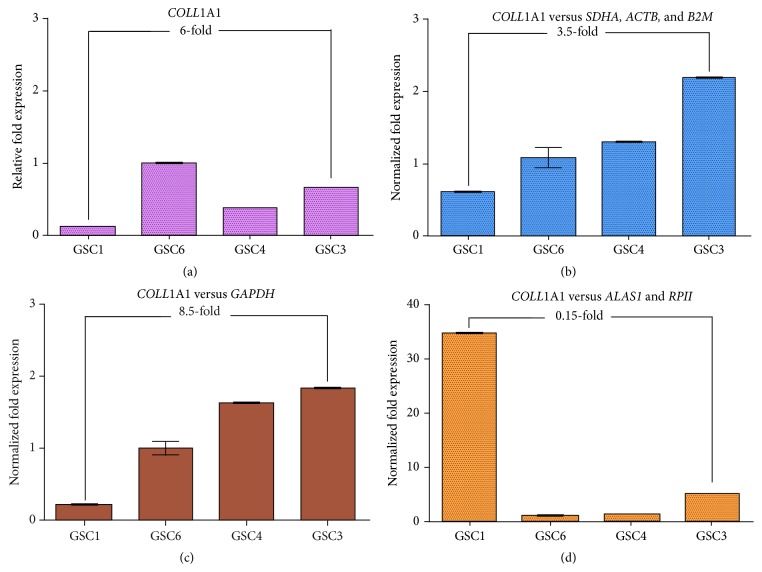
Effect of the choice of stable HKGs in the study of four samples of GSC. The relative fold expression (a) and the normalized fold expression of* COLL*1A1 of 4 samples of GSC. The normalization was performed with* SDHA/B2M/ACTB*, the most stable genes (b),* GAPDH*, the most usually used (c), and* ALAS1*/*RPII*, the least stable genes (d). Error bars expressed the standard deviation of the mean.

The first analysis compared four random samples of GSC, GSC1, GSC3, GSC4, and GSC6, and their expression of a target gene,* Coll*agen 1 alpha 1 (*COLL*1A1). The relative fold expression with no normalization of this gene showed differences between the four samples: GSC6 had the highest relative fold expression (1.00 ± 0.028), followed by GSC3 (0.66 ± 0.001), GSC4 (0.38 ± 0.001), and GSC1 (0.11 ± 0.003). GSC3 had a 6-fold higher expression than GSC1 (Figure 7(a)). Data were then normalized with three groups of genes:* SDHA*,* ACTB*, and* B2M* were the most stable genes as suggested by our present study,* GAPDH* was the most commonly used reference gene, and* ALAS1* and* RPS18* were the least stable ones according to our results.

The normalization with* SDHA*,* ACTB*, and* B2M* showed a different ranking: GSC3 (2.21 ± 0.021) had the highest normalized fold expression, followed by GSC4 (1.31 ± 0.015), GSC6 (1.09 ± 0.141), and GSC1 (0.62 ± 0.023). In this ranking, GSC3 had a 3.5-fold higher expression than GSC1 (Figure 7(b)). When normalized with* GAPDH*, the ranking was the same as the normalization with the stable HKGs, but GSC3 (1.83 ± 0.020) had an 8.5-fold higher expression than GSC1 (0.21 ± 0.014) (Figure 7(c)). At last, the normalization with* ALAS1* and* RPII* showed a different ranking, unlike the former analyses, GSC1 (38.98 ± 0.295) had the highest normalized expression, followed by GSC3 (5.02 ± 0.097), GSC4 (1.37 ± 0.070), and GSC6 (1.01 ± 0.300). GSC1 had a 0.15-fold expression as compared to GSC3.

**Figure 8:**
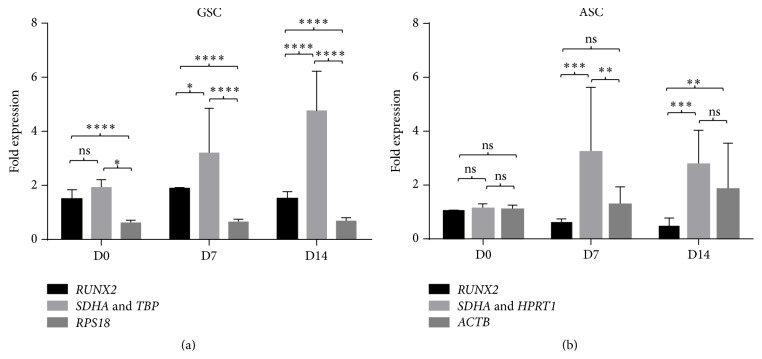
Effect of the choice of stable HKGs in the study of dGSC (*n* = 6) (a) and ASC (*n* = 6) (b). The normalization of fold expression of an osteogenic marker,* RUNX2*, was performed on D0, D7, and D14. As calibrators: for dGSCs:* TBP*/*SDHA* the most stable HKGs and* RPS18* the least stable one. For ASCs:* SDHA*/*HPRT1* the most stable HKGs and* ACTB* the least stable one. *p* values have been obtained with unpaired *t*-test. ^*∗∗∗∗*^ *p* ≤ 0.0001, ^*∗∗∗*^ *p* ≤ 0.001, and ^*∗∗*^ *p* ≤ 0.01 and “ns” stands for *p* ≥ 0.05.

The second analysis was performed on samples of dGSCs (*n* = 6) and compared to ASCs (*n* = 6) after osteogenic differentiation. Expression level of* RUNX2* was assessed on different time points: day 0 (D0), day 7 (D7) and day 14 (D14) after osteogenic differentiation, confirmed by the microscopic observation of mineral nodules formation and Alizarin Red S staining (Supplementary Material 3). The normalization was performed against the two most stable reference genes and compared to the least stable one as shown in Figure 8. For dGSCs, the normalization to* SDHA* and* TBP* showed a significant increase of* RUNX2* expression at D7 and D14 when compared to the relative gene expression (Figure 8(a)). When data were normalized with* RPS18*, the least stable reference gene for dGSCs, there was a decrease in the expression of* RUNX2*, with a statistically significant difference when compared to* SDHA* and* TBP*. Concerning ASCs, the normalization with* SDHA* and* HPRT1* showed also an increasing expression level of* RUNX2* at D7 and D14 and an insignificant increase at D7 of this target gene when normalized with* ACTB*, the least stable reference gene (Figure 8(b)). This confirms the importance and the dramatic effect of the choice of suitable HKGs for GSCs before and after osteogenic differentiation.

## 4. Discussion

This study was performed to confirm previously well-recognized properties of GSCs [1] as well as their osteogenic potential [2]. GSCs might be an interesting tool in human cell therapy and especially in bone regeneration of craniofacial bones that share the same embryonic origin. Our study compared human GSCs to adipose-derived mesenchymal stem cells (ASCs), as they have been well studied in their differentiation capacities, thoroughly highlighted* in vitro* and* in vivo* [25]. The osteogenic differentiation capacity of GSCs needs to be supported by q-PCR, which measures the relative expression of genes implicated in osteogenic differentiation. q-PCR is a very accurate method for gene expression analysis; however, data analyses are often variable. This is due to many factors linked to each step of the investigations, from harvesting cells to cDNA synthesis. That is why q-PCR results have to be normalized. The validation of stable and adequate reference genes, to which data are normalized, is the most commonly used method and may allow bypassing variability factors. The choice of a reference gene must be accurate, because any improper HKG may give false interpretation of q-PCR results [26].* GAPDH* is the most used reference gene, but recent studies showed that this gene is not suitable for all tissues or cell types or experimental settings [6]. An ideal HKG is expected to have a constant level of expression in all cell types, at all-time points, and under different experimental conditions. Studies have been carried out to reach a “universal HKG” in all species but, to our knowledge, not one was found [13]. To solve this problem of choosing suitable HKGs, many algorithms were generated by mathematicians and statisticians in order to select accurately the most stable reference genes.

In this work, we aimed to select the most appropriate HKGs to study human GSCs and their osteogenic differentiation* in vitro* in order to use them for clinical application. 10 reference genes were studied on GSCs, dGSCs, and ASCs (mesenchymal stem cells, with known osteogenic capacity) and analysed by four algorithms: Δ*Ct comparative method, GeNorm, BestKeeper*, and* NormFinder*.

Experiments were rigorously conducted to obtain comparable and reproducible results; a special attention was given to prepare accurately with the same conditions growing and differentiation media while culturing different stem cells. The used RNA extraction kit was appropriate to cell culture and the products were carefully used at the adequate amount corresponding to cells number. RT q-PCR reactions were performed according to the MIQE Guidelines (Supplementary Material 4) [7]. Consequently, in our study, despite a slight dissimilarity in the ranking of HKGs among the four algorithms, results showed a highly homogenous reproducibility; indeed, a similar tendency was found for the three most stable genes and an overall comparable order of genes.

Δ*Ct comparative method* and* GeNorm* were used for intergene study; Δ*Ct comparative method* identifies the most reliable reference gene as the one that varies the less when compared to all the other genes among all the sample. This method needs no high-level mathematical methodology and is ideal for the nonspecialist to determine the best reference genes [13].* GeNorm* software not only defines the optimal combination of genes or the ideal “pair of genes” with low variability, but also proposes the minimal number of stable HKGs. These two methods can be used if the amount of the starting material of studied samples is not enough to study many HKGs, because they are minimally affected by expression intensity [23]. However, they do not take into account coregulated genes and if used alone they may lead to errors. These algorithms showed the same ranking of the ten reference genes in the three groups.* SDHA, ACTB*, and* B2M* were the most stable to study GSCs;* TBP, SDHA*, and* ALAS1* for dGSCs; and* SDHA, HPRT1*, and* TBP* for ASCs.

For more accuracy, we used* BestKeeper* and* NormFinder,* which study also intragene variability.* BestKeeper* is an interesting tool, because it considers not only intra- and intergroup variation, but also sample integrity. Our samples seemed to have conserved their integrity when applying this analysis throughout all the groups (22 samples of GSCs, dGSCs, and ASCs).* NormFinder* is considered to be an extra control for* GeNorm* [22] because it uses a different algorithm and relies on calculating the stability value for each HKG. These two methods seem to be very sensitive to the size of samples and to expression intensity. The ranking of stable reference genes was the same as Δ*Ct comparative method* and* GeNorm* for GSCs but slightly different for dGSCs and ASCs.* BestKeeper* ranked* B2M*,* SDHA*, and* HPRT1* as the most stable for* dGSCs* and* HPRT1*,* SDHA*, and* RPS18* for ASCs. The least stable genes were ranked differently than Δ*Ct comparative method* and* GeNorm* for the three groups of samples.* NormFinder* ranking was less different from the two precedent algorithms, and the only difference was noticed on ASCs ranking.* HPRT1* was ranked 8th rather than with the three most stable reference genes. This can be due to the decreased sample size of ASC (*n* = 6) which may affect* BestKeeper* and* NormFinder* analyses.

Ideally, the ranking of stable HKGs must rely on the results found by the four methods, because the reproducibility and accuracy increase, as the mathematical and statistical bases are different.* RefFinder* by calculating the geometric mean of overall ordering may help us to identify more easily the stable reference genes; however, it does not take into account q-PCR efficiencies of different primers and only uses Ct values.

Finally, when we analysed each group of samples separately, we found that* SDHA*,* B2M*, and* ACTB* were ranked as the most stable for GSCs;* TBP, SDHA*, and* ALAS1* for dGSCs; and* SDHA* and* HPRT1* for ASCs.

The analysis of gene expression for GSCs either differentiated or not showed that the normalization with random reference genes leads to errors. Our results regarding osteogenic differentiation of GSCs were in agreement with previous publications;* TBP* was found as a suitable reference gene to study osteogenic differentiation in stem cells [24]. Regardless of the origin of stem cells and the stage of differentiation,* SDHA* seems to be a good reference gene, as it was stable in all conditions but is not enough alone to study GSC. Ribosomal genes were the least stable, but they might be useful in other conditions [21].

This present study provides methods to determine suitable reference genes. These tools are crucial to studying further the GSC properties and compare the stem cell lineages. Indeed, the osteogenic differentiation can be thoroughly investigated under different conditions (conditioned media, biomaterials, etc.) in order to use GSCs in human bone regeneration.

## 5. Conclusion

Regardless of the algorithm used in this study, all of the software used has ranked the same set of reference genes as the most stable. Finally, based on our results, we recommend the use of* SDHA*,* B2M*, and* ACTB* for GSCs;* TBP*,* SDHA*, and* ALAS1* for dGSCs; and* SDHA* and* HPRT1* for ASCs study. We stress on the importance to select the most suitable HKGs before conducting a study of q-PCR of any stem cell type.

## Supplementary Material

Supplementary Material 1: Three tables showing normalized values (R) of the three groups of samples GSCs, dGSCs and ASCs with the ten studied HKGs. R were calculated using formula and depend on E the efficiency of each primer (1).
Supplementary Material 2: Two tables describing the Δ*Ct comprative method* for dGSCs and ASCs and the ranking of the ten HKGs according to StdDev mean of each gene.
Supplementary Material 3: Mineral nodules were observed for dGSCs at different time points D7, D14 and D21. This confirmed the osteogenic differentiation in accordance with the increase of RUNX2 gene expression. Alizarin Red S staining at D21 confirmed the calcium content of these nodules.
Supplementary Material 4: MIQE checklist was almost respected by the different steps of our study, from collecting samples to gene expression analyses, which is in accordance with the accuracy of our results.

## Figures and Tables

**Table 1:** 10 candidate housekeeping genes analysed.

Gene symbol	Gene name	Gene accession number	Primer sequence (F/R)	*E* (%)	Amplicon size (bp)
*TBP*	TATA box binding protein	NM_003194.4	F: CAC GAA CCA CGG CAC TGA TT	94,2	88
			R: TTT TCT TGC TGC CAG TCT GGA C		

*SDHA*	Succinate dehydrogenase complex, subunit A, flavoprotein	NM_004168.3	F: AGC AAG CTC TAT GGA GAC CT	88,4	199
			R: TAA TCG TAC TCA TCA ATC CG		

*HPRT1*	Hypoxanthine phosphoribosyltransferase I	NM_000194.2	F: TGT TGG ATT TGA AAT TCC AGA CAA G	90,5	107
			R: CTT TTC CAG TTT CAC TAA TGA CAC AA		

*GAPDH*	Glyceraldehyde-3-phosphate dehydrogenase	NM_002046.5	F: CTT TGT CAA GCT CAT TTC CTG GTA	82,5	70
			R: GGC CAT GAG GTC CAC CA		

*RPS18*	Ribosomal protein S18	NM_022551.2	F: AGC TTG TTG TCC AGA CCA TT	86,7	188
			R: TGA GGA AAG CAG ACA TTG AC		

*ALAS1*	5′-Aminolevulinate synthase 1	NM_000688.5	F: AAC TTC CCA AAA TCT GTT TC	88,8	158
			R: GGT GAT GAG GGA GTC TGA AT		

*ACTB*	Actin, beta	NM_001101.3	F: CTG TGG CAT CCA CGA AAC	96	88
			R: CAG ACA GCA CTG TGT TGG		

*B2M*	Beta-2-microglobulin	NM_004048.2	F: CAG CAT CAT GGA GGT TTG AA	92	178
			R: TGG AGA CAG CAC TCA AAG TA		

*UBC*	Ubiquitin C	NM_021009.6	F: GTG GCA CAG CTA GTT CCG T	103	96
			R: CTT CAC GAA GAT CTG CAT TGT CA		

*RPII*	50S ribosomal protein L9	NM_031420.3	F: CTT CAC GGT GCT GGG CAT T	95	240
			R: GTG CGG CTG CTT CCA TAA		

**Table 2:** The 4 algorithms used in the study.

Analysis method	PCR values	Stability values	Type of analysis
The ΔCt comparative method	Ct	StdDev mean	Intergene

GeNorm	*R*	*M*, NF	Intergene One HKG → all other HKGs Stable HKGs: lowest variability: low *M*

*BestKeeper*	Ct	SD, *r*	Intra- and intergene Pairwise correlation analysis → BestKeeper index Stable HKGs: low SD and high *r*

NormFinder	*R*	*ρ*	Intra- and intergene; stable HKGs: least intra- and intergroup variations

**Table 3:** The ΔCt comparative method analysis for GSCs.

Gene versus gene	Mean ΔCt	StdDev	Mean StdDev	Gene versus Gene	Mean ΔCt	StdDev	Mean StdDev
*TBP* versus *SDHA*	3,73	0,84	** 1.05**	*ALAS1* versus *TBP*	0,56	1,51	** 1.27**
*TBP* versus *HPRT1*	1,85	0,86		*ALAS1* versus *SDHA*	−3,18	1,02	
*TBP* versus *GAPDH*	8,48	0,92		*ALAS1* versus *HPRT1*	−1,30	1,87	
*TBP* versus *RPS18*	8,99	1,00		*ALAS1* versus *GAPDH*	−7,93	1,27	
*TBP* versus *ALAS1*	0,56	1,51		*ALAS1* versus *RPS18*	−8,43	1,37	
*TBP* versus *ACTB*	8,67	0,85		*ALAS1* versus *ACTB*	8,12	1,20	
*TBP* versus *B2M*	9,29	0,88		*ALAS1* versus *B2M*	8,73	0,99	
*TBP* versus *UBC*	−1,68	0,64		*ALAS1* versus *UBC*	−2,24	1,36	
*TBP* versus *RPII*	0,99	1,99		*ALAS1* versus *RPII*	0,43	0,85	

*SDHA* versus *TBP*	3,73	0,84	** 0.97**	*ACTB* versus *TBP*	8,67	0,85	** 0.98**
*SDHA* versus *HPRT1*	−1,88	1,36		*ACTB* versus *SDHA*	4,94	0,63	
*SDHA* versus *GAPDH*	4,75	0,75		*ACTB* versus *HPRT1*	6,82	1,01	
*SDHA* versus *RPS18*	5,25	1,00		*ACTB* versus *GAPDH*	0,19	0,93	
*SDHA* versus *ALAS1*	−3,18	1,02		*ACTB* versus *RPS18*	−0,31	1,18	
*SDHA* versus *ACTB*	4,94	0,63		*ACTB* versus *ALAS1*	8,12	1,20	
*SDHA* versus *B2M*	5,56	0,90		*ACTB* versus *B2M*	0,62	0,83	
*SDHA* versus *UBC*	−5,41	0,89		*ACTB* versus *UBC*	−10,35	0,67	
*SDHA* versus *RPII*	−2,75	1,33		*ACTB* versus *RPII*	−7,69	1,54	

*HPRT1* versus *TBP*	1,85	0,86	** 1.37**	*B2M* versus *TBP*	9,29	0,88	** 0.98**
*HPRT1* versus *SDHA*	−1,88	1,36		*B2M* versus *SDHA*	5,56	0,90	
*HPRT1* versus *GAPDH*	6,63	1,42		*B2M* versus *HPRT1*	7,44	1,27	
*HPRT1* versus *RPS18*	7,13	1,23		*B2M* versus *GAPDH*	0,81	0,72	
*HPRT1* versus *ALAS1*	−1,30	1,87		*B2M* versus *RPS18*	0,30	1,09	
*HPRT1* versus *ACTB*	6,82	1,01		*B2M* versus *ALAS1*	8,73	0,99	
*HPRT1* versus *B2M*	7,44	1,27		*B2M* versus *ACTB*	0,62	0,83	
*HPRT1* versus *UBC*	−3,53	1,03		*B2M* versus *UBC*	−10,97	0,70	
*HPRT1* versus *RPII*	−0,87	2,41		*B2M* versus *RPII*	−8,30	1,47	

*GAPDH* versus *TBP*	8,48	0,92	** 1.06**	*UBC* versus *TBP*	−1,68	0,64	** 1.02**
*GAPDH* versus *SDHA*	4,75	0,75		*UBC* versus *SDHA*	−5,41	0,89	
*GAPDH* versus *HPRT1*	6,63	1,42		*UBC* versus *HPRT1*	−3,53	1,03	
*GAPDH* versus RSP18	0,50	1,16		*UBC* versus *GAPDH*	−10,16	0,86	
*GAPDH* versus *ALAS1*	−7,93	1,27		*UBC* versus *RPS18*	−10,66	1,39	
*GAPDH* versus *ACTB*	0,19	0,93		*UBC* versus *ALAS1*	−2,24	1,36	
*GAPDH* versus *B2M*	0,81	0,72		*UBC* versus *ACTB*	−10,35	0,67	
*GAPDH* versus *UBC*	−10,16	0,86		*UBC* versus *B2M*	−10,97	0,70	
*GAPDH* versus *RPII*	−7,50	1,54		*UBC* versus *RPII*	2,67	1,68	

*RPS18* versus *TBP*	8,99	1,00	** 1.27**	*RPII* versus *TBP*	0,99	1,99	** 1.64**
*RPS18* versus *SDHA*	5,25	1,00		*RPII* versus *SDHA*	−2,75	1,33	
*RPS18* versus *HPRT1*	7,13	1,23		*RPII* versus *HPRT1*	−0,87	2,41	
*RPS18* versus *GAPDH*	0,50	1,16		*RPII* versus *GAPDH*	−7,50	1,54	
*RPS18* versus *ALAS1*	−8,43	1,37		*RPII* versus *RPS18*	−8,00	2,00	
*RPS18* versus ACTIN	−0,31	1,18		*RPII* versus *ALAS1*	0,43	0,85	
*RPS18* versus *B2M*	0,30	1,09		*RPII* versus *ACTB*	−7,69	1,54	
*RPS18* versus *UBC*	−10,66	1,39		*RPII* versus *B2M*	−8,30	1,47	
*RPS18* versus *RPII*	−8,00	2,00		*RPII* versus *UBC*	2,67	1,68	

**Table 4:** *GeNorm* analysis.

Rank	GSCs (*n* = 8)		dGSCs (*n* = 8)		ASCs (*n* = 6)	
	Gene	*M* values	Gene	*M* values	Gene	*M* values
1	*SDHA*	0.892	*TBP*	0.656	*SDHA*	0.619
2	*B2M*	0.919	*SDHA*	0.677	*HPRT1*	0.656
3	*ACTB*	0.924	*ALAS1*	0.729	*TBP*	0.717
4	*UBC*	0.964	*RPII*	0.771	*RPS18*	0.741
5	*GAPDH*	0.968	*B2M*	0.777	*UBC*	0.778
6	*TBP*	0.985	*ACTB*	0.729	*GAPDH*	0.786
7	*HPRT1*	1.294	*GAPDH*	0.849	*B2M*	0.806
8	*RPS18*	1.162	*HPRT1*	0.849	*RPII*	0.997
9	*ALAS1*	1.163	*UBC*	0.858	*ALAS1*	1.081
10	*RPII*	1.588	*RPS18*	0.925	*ACTB*	1.491

**(a):** 

GSCs (*n* = 8)	*SDHA*	*B2M*	*ALAS1*	*RPII*	*ACTB*	*GAPDH*
Coefficient of correlation (*r*)	0.938	0.861	0.894	0.886	0.841	0.799
*p* value	0.001	0.006	0.003	0.003	0.007	0.017

**(b):** 

dGSCs (*n* = 8)	*RPII*	*B2M*	*SDHA*	*UBC*	*HPRT1*	*TBP*
Coefficient of correlation (*r*)	0.900	0.823	0.812	0.810	0.776	0.752
*p* value	0.002	0.012	0.014	0.015	0.024	0.031

**(c):** 

ASCs (*n* = 6)	*HPRT1*	*SDHA*	*RPS18*	*RPII*
Coefficient of correlation (*r*)	0.914	0.893	0.864	0.813
*p* value	0.011	0.017	0.027	0.049

**Table 6:** *NormFinder* analysis.

Rank	GSCs (*n* = 8)		dGSCs (*n* = 8)		ASCs (*n* = 6)	
	Gene	*ρ* values	Gene	*ρ* values	Gene	*ρ* values
1	*SDHA*	0.248	*TBP*	0.222	*SDHA*	0.063
2	*B2M*	0.3	*SDHA*	0.234	*TBP*	0.224
3	*ACTB*	0.317	*ALAS1*	0.335	*RPS18*	0.246
4	*UBC*	0.388	*RPII*	0.364	*UBC*	0.257
5	*GAPDH*	0.401	*B2M*	0.380	*GAPDH*	0.42
6	*TBP*	0.453	*ACTB*	0.411	*B2M*	0.464
7	*HPRT1*	0.608	*GAPDH*	0.454	*RPII*	0.487
8	*ALAS1*	0.616	*HPRT1*	0.457	*HPRT1*	0.514
9	*RPS18*	0.78	*UBC*	0.472	*ALAS1*	0.671
10	*RPII*	1.025	*RPS18*	0.528	*ACTB*	1.1

**Table 7:** Final ranking of HKGs.

Algorithm	GSC	dGSC	ASC
ΔCt comparative method	*SDHA/ACTB/B2M*	*TBP/SDHA/ALAS1*	*SDHA/HPRT1*

GeNorm	*SDHA/ACTB/UBC *	*TBP/ALAS1/SDHA*	*GAPDH/B2M*
	*SDHA/B2M/ACTB *	*TBP/SDHA/ALAS1 *	*HPRT1/SDHA*
	*3 genes*	*3 genes*	*2 genes*

BestKeeper	*SDHA/B2M/ACTB*	*B2M/SDHA/HPRT1*	*HPRT1/SDHA*

NormFinder	*SDHA/B2M/ACTB*	*TBP/SDHA/ALAS1*	*SDHA/TBP/RPS18*

RefFinder	*UBC/ACTB/SDHA*	*TBP/SDHA/ACTB*	*HPRT1/SDHA*

Selected genes	***SDHA/ACTB/B2M***	***TBP/SDHA***	***HPRT1/SDHA***

## Abbreviations

ASCs: Adipose-derived stem cells
dGSCs: Differentiated gingival stem cells
GSCs: Gingival stem cells
*E*: Efficiency
MIQE: Minimum Information for Publication of Quantitative Real-Time PCR Experiments
qPCR: Quantitative Polymerase Chain Reaction
*R*: Relative expression.

## References

[1] Fournier B. P. J., Ferre F. C., Couty L. (2010). Multipotent progenitor cells in gingival connective tissue. *Tissue Engineering Part A*.

[2] Fournier B. P. J., Larjava H., Häkkinen L. (2013). Gingiva as a source of stem cells with therapeutic potential. *Stem Cells and Development*.

[3] Ferré F. C., Larjava H., Loison-Robert L.-S. (2014). Formation of cartilage and synovial tissue by human gingival stem cells. *Stem Cells and Development*.

[4] Leucht P., Kim J.-B., Amasha R., James A. W., Girod S., Helms J. A. (2008). Embryonic origin and Hox status determine progenitor cell fate during adult bone regeneration. *Development*.

[5] Zhao D., Cui D., Wang B. (2012). Treatment of early stage osteonecrosis of the femoral head with autologous implantation of bone marrow-derived and cultured mesenchymal stem cells. *Bone*.

[6] Bustin S. A. (2002). Quantification of mRNA using real-time reverse transcription PCR (RT-PCR): trends and problems. *Journal of Molecular Endocrinology*.

[7] Bustin S. A., Benes V., Garson J. A. (2009). The MIQE guidelines: minimum information for publication of quantitative real-time PCR experiments. *Clinical Chemistry*.

[8] Van Peer G., Mestdagh P., Vandesompele J. (2012). Accurate RT-qPCR gene expression analysis on cell culture lysates. *Scientific Reports*.

[9] Kozera B., Rapacz M. (2013). Reference genes in real-time PCR. *Journal of Applied Genetics*.

[10] Johnson G., Nour A. A. B., Nolan T., Huggett J., Bustin S. (2014). Minimum information necessary for quantitative real-time PCR experiments. *Methods in Molecular Biology*.

[11] Andersen C. L., Jensen J. L., Ørntoft T. F. (2004). Normalization of real-time quantitative reverse transcription-PCR data: A model-based variance estimation approach to identify genes suited for normalization, applied to bladder and colon cancer data sets. *Cancer Research*.

[12] Pfaffl M. W., Tichopad A., Prgomet C., Neuvians T. P. (2004). Determination of stable housekeeping genes, differentially regulated target genes and sample integrity: BestKeeper—excel-based tool using pair-wise correlations. *Biotechnology Letters*.

[13] Silver N., Best S., Jiang J., Thein S. L. (2006). Selection of housekeeping genes for gene expression studies in human reticulocytes using real-time PCR. *BMC Molecular Biology*.

[14] Vandesompele J., De Preter K., Pattyn F. (2002). Accurate normalization of real-time quantitative RT-PCR data by geometric averaging of multiple internal control genes. *Genome Biology*.

[15] Ragni E., Viganò M., Rebulla P., Giordano R., Lazzari L. (2013). What is beyond a qRT-PCR study on mesenchymal stem cell differentiation properties: how to choose the most reliable housekeeping genes. *Journal of Cellular and Molecular Medicine*.

[16] Rotter N., Oder J., Schlenke P. (2008). Isolation and characterization of adult stem cells from human salivary glands. *Stem Cells and Development*.

[17] Li D.-R., Cai J.-H. (2012). Methods of isolation, expansion, differentiating induction and preservation of human umbilical cord mesenchymal stem cells. *Chinese Medical Journal*.

[18] Hilkens P., Gervois P., Fanton Y. (2013). Effect of isolation methodology on stem cell properties and multilineage differentiation potential of human dental pulp stem cells. *Cell and Tissue Research*.

[19] Chevallier N., Anagnostou F., Zilber S. (2010). Osteoblastic differentiation of human mesenchymal stem cells with platelet lysate. *Biomaterials*.

[20] Walker N. J. (2002). A technique whose time has come. *Science*.

[21] Chen D., Pan X., Xiao P., Farwell M. A., Zhang B. (2011). Evaluation and identification of reliable reference genes for pharmacogenomics, toxicogenomics, and small RNA expression analysis. *Journal of Cellular Physiology*.

[22] Van Hiel M. B., Van Wielendaele P., Temmerman L. (2009). Identification and validation of housekeeping genes in brains of the desert locust *Schistocerca gregaria* under different developmental conditions. *BMC Molecular Biology*.

[23] Mehta R., Birerdinc A., Hossain N. (2010). Validation of endogenous reference genes for qRT-PCR analysis of human visceral adipose samples. *BMC Molecular Biology*.

[24] Rauh J., Jacobi A., Stiehler M. (2015). Identification of stable reference genes for gene expression analysis of three-dimensional cultivated human bone marrow-derived mesenchymal stromal cells for bone tissue engineering. *Tissue Engineering Part C: Methods*.

[25] Grayson W. L., Bunnell B. A., Martin E., Frazier T., Hung B. P., Gimble J. M. (2015). Stromal cells and stem cells in clinical bone regeneration. *Nature Reviews Endocrinology*.

[26] Dheda K., Huggett J. F., Chang J. S. (2005). The implications of using an inappropriate reference gene for real-time reverse transcription PCR data normalization. *Analytical Biochemistry*.

