# Supplementary material for: Validation of Housekeeping Genes to Study Human Gingival Stem Cells and Their In Vitro Osteogenic Differentiation Using Real-Time RT-qPCR
Source: Stem Cells Int. 2015 Dec 30;2016:6261490. doi: 10.1155/2016/6261490 (PMC4736224; doi:10.1155/2016/6261490)
Supplement: Supplementary file 1 — Supplementary Material 1: Three tables showing normalized values (R) of the three groups of samples GSCs, dGSCs and ASCs with the ten studied HKGs. R were calculated using formula and depend on E the efficiency of each primer (1). Supplementary Material 2: Two tables describing the ΔCt comprative method for dGSCs and ASCs and the ranking of the ten HKGs according to StdDev mean of each gene. Supplementary Material 3: Mineral nodules were observed for dGSCs at different time points D7, D14 and D21. This confirmed the osteogenic differentiation in accordance with the increase of RUNX2 gene expression. Alizarin Red S staining at D21 confirmed the calcium content of these nodules. Supplementary Material 4: MIQE checklist was almost respected by the different steps of our study, from collecting samples to gene expression analyses, which is in accordance with the accuracy of our results. [file 6261490.f1.pdf]

Supplementary material 1: Normalized values (R) of GSC dGSC and ASC samples for the 10 HKGs.

Table 1: The normalized values $R = E_{HKG}^{\Delta Ct(\text{Calibrator sample- studied sample})}$ for GSCs

GSCs n=8	TBP	SDHA	HPRT1	GAPDH	RPS18	ALAS1	ACTIN	B2M	UBC
GSC1	1	0,460	0,852	0,490	0,469	0,080	0,306	0,221	0,283
GSC2	0,284	0,096	0,466	0,219	0,090	0,077	0,103	0,186	0,210
GSC3	1	0,296	0,789	0,336	0,317	0,272	0,306	0,549	0,473
GSC4	0,654	0,416	1	0,224	0,835	0,493	0,329	0,352	0,228
GSC5	0,820	1	0,558	0,830	0,311	0,939	1	0,936	1
GSC6	0,646	0,421	0,702	0,887	1	0,487	0,362	1	0,301
GSC7	0,936	0,802	0,272	1	0,652	1	0,253	0,694	0,407
GSC8	0,629	0,711	0,852	0,698	0,531	0,729	0,637	0,457	0,359

Table 2: The normalized values $R = E_{HKG}^{\Delta Ct(\text{Calibrator sample- studied sample})}$ for dGSCs

dGSCs n=8	TBP	SDHA	HPRT1	GAPDH	RPS18	ALAS1	ACTIN	B2M	UBC
dGSC1	1	0,676	0,725	0,317	1	1	0,390	0,472	0,928
dGSC2	0,573	0,506	0,431	0,605	0,452	0,539	0,531	0,791	1
dGSC3	0,288	0,252	0,144	0,370	0,422	0,176	0,233	0,196	0,151
dGSC4	0,460	1	1	1	0,377	0,378	0,411	1	0,695
dGSC5	0,565	0,364	0,377	0,517	0,335	0,429	0,624	0,414	0,517
dGSC6	0,476	0,457	0,594	0,698	0,184	0,478	0,379	0,334	0,338
dGSC7	0,543	0,395	0,789	0,569	0,298	0,369	1	0,919	0,248
dGSC8	0,390	0,388	0,852	0,422	0,455	0,266	0,500	0,741	0,232

Table 3: The normalized values $R = E_{HKG}^{\Delta Ct(\text{Calibrator sample- studied sample})}$ for ASCs

**ASCs n=8**	TBP	SDHA	HPRT1	GAPDH	RPS18	ALAS1	ACTIN	B2M	UBC
ASC1	1	1	0,333	1	0,417	1	0,044	1	0,782
ASC2	0,522	0,847	0,554	0,993	1	0,351	0,244	0,825	0,569
ASC3	0,809	0,830	0,422	0,476	0,620	0,540	1	0,406	0,967
ASC4	0,295	0,457	0,192	0,612	0,260	0,122	0,225	0,425	0,454
ASC5	0,422	0,667	0,259	0,616	0,482	0,155	0,356	0,442	1
ASC6	0,543	0,577	1	0,577	0,482	0,183	0,370	0,358	0,825

Supplementary material 2: Ranking of 10 HKG in dGSC and ASC with CT comparative method.

Table 1: The ∆ Ct Comparative Method Analysis for dGSCs

Gene vs Gene	Mean ∆ Ct	StdDev	Mean StdDev	Gene vs Gene	Mean ∆ Ct	StdDev	Mean StdDev
TBP vs *SDHA*	4,31	0,60		*ALAS1* vs *TBP*	1,49	0,32	
TBP vs *HPRT1*	1,15	0,87		*ALAS1* vs *SDHA*	-2,82	0,67	
TBP vs *GAPDH*	8,39	0,88		*ALAS1* vs *HPRT1*	0,34	0,94	
TBP vs *RPS18*	9,06	0,69		*ALAS1* vs *GAPDH*	-6,9	1,03	
TBP vs *ALAS1*	1,49	0,32	**0,69**	*ALAS1* vs *RPS18*	-7,57	0,87	**0,78**
TBP vs *ACTB*	8,14	0,67		*ALAS1* vs *ACTB*	6,65	0,90	
TBP vs *B2M*	9,35	0,87		*ALAS1* vs *B2M*	7,86	1,02	
TBP vs *UBC*	-0,74	0,63		*ALAS1* vs *UBC*	-2,23	0,53	
TBP vs *RPII*	2,41	0,70		*ALAS1* vs *RPII*	0,92	0,78	
SDHA vs *TBP*	-4,31	0,60		*ACTB* vs *TBP*	8,14	0,67	
SDHA vs *HPRT1*	-3,16	0,69		*ACTB* vs *SDHA*	3,83	0,89	
SDHA vs *GAPDH*	4,07	0,63		*ACTB* vs *HPRT1*	6,99	0,85	
SDHA vs *RPS18*	4,75	0,88		*ACTB* vs *GAPDH*	-0,25	0,77	
SDHA vs *ALAS1*	-2,82	0,67	**0,72**	*ACTB* vs *RPS18*	-0,92	1,09	**0,85**
SDHA vs *ACTB*	3,83	0,89		*ACTB* vs *ALAS1*	6,65	0,90	
SDHA vs *B2M*	5,04	0,71		*ACTB* vs *B2M*	1,21	0,64	
SDHA vs *UBC*	-5,06	0,62		*ACTB* vs *UBC*	-8,88	1,06	
SDHA vs *RPII*	-1,90	0,78		*ACTB* vs *RPII*	-5,73	0,76	
HPRT1 vs *TBP*	1,15	0,87		*B2M* vs *TBP*	9,35	0,87	
HPRT1 vs *SDHA*	-3,16	0,69		*B2M* vs *SDHA*	5,04	0,71	
HPRT1 vs *GAPDH*	7,23	0,93		*B2M* vs *HPRT1*	8,20	0,59	
HPRT1 vs *RPS18*	7,91	1,22		*B2M* vs *GAPDH*	0,96	0,78	
HPRT1 vs *ALAS1*	0,34	0,94	**0,91**	*B2M* vs *RPS18*	0,29	1,12	**0,82**
HPRT1 vs *ACTB*	6,99	0,85		*B2M* vs *ALAS1*	7,86	1,02	
HPRT1 vs *B2M*	8,20	0,60		*B2M* vs *ACTB*	1,21	0,64	
HPRT1 vs *UBC*	-1,90	1,08		*B2M* vs *UBC*	-10,09	0,98	
HPRT1 vs *RPII*	1,26	0,99		*B2M* vs *RPII*	-6,94	0,67	
GAPDH vs *TBP*	8,39	0,88		*UBC* vs *TBP*	-0,74	0,63	
GAPDH vs *SDHA*	4,08	0,63		*UBC* vs *SDHA*	-5,06	0,62	
GAPDH vs *HPRT1*	7,24	0,93		*UBC* vs *HPRT1*	-1,90	1,08	
GAPDH vs RSP18	0,67	1,25	**0,92**	*UBC* vs *GAPDH*	-9,13	0,99	**0,82**
GAPDH vs *ALAS1*	-6,9	1,03		*UBC* vs *RPS18*	-9,80	0,94	
GAPDH vs *ACTB*	-0,25	0,77		*UBC* VS *ALAS1*	-2,23	0,53	
GAPDH vs *B2M*	0,96	0,78		*UBC* vs *ACTB*	-8,88	1,06	

GAPDH vs *UBC*	-9,13	0,99		*UBC* vs *B2M*	-10,09	0,98	
GAPDH vs *RPII*	-5,99	1,02		*UBC* vs *RPII*	3,15	0,59	
RPS18 vs *TBP*	9,06	0,69		*RPII* vs *TBP*	2,41	0,70	
RPS18 vs *SDHA*	4,75	0,88		*RPII* vs *SDHA*	-1,90	0,78	
RPS18 vs *HPRT1*	7,91	1,22		*RPII* vs *HPRT1*	1,26	0,99	
RPS18 vs *GAPDH*	0,67	1,25	**1,00**	*RPII* vs *GAPDH*	-5,98	1,02	**0,80**
RPS18 vs *ALAS1*	-7,57	0,87		*RPII* vs *RPS18*	-6,65	0,95	
RPS18 vs ACTIN	-0,92	1,09		*RPII* vs *ALAS1*	0,92	0,78	
RPS18 vs *B2M*	0,29	1,12		*RPII* vs *ACTB*	-5,73	0,76	
RPS18 vs *UBC*	-9,80	0,94		*RPII* vs *B2M*	-6,94	0,67	
RPS18 vs *RPII*	-6,65	0,95		*RPII* vs *UBC*	3,15	0,59	

Table 2: The ∆ Ct Comparative Method Analysis for ASCs

Gene vs Gene	Mean ∆ Ct	StdDev	Mean StdDev	Gene vs Gene	Mean ∆ Ct	StdDev	Mean StdDev
TBP vs *SDHA*	4,40	0,36		*ALAS1* vs *TBP*	1,47	0,64	
TBP vs *HPRT1*	0,82	0,57		*ALAS1* vs *SDHA*	-2,93	0,85	
TBP vs *GAPDH*	8,31	0,71		*ALAS1* vs *HPRT1*	0,65	1,02	
TBP vs *RPS18*	8,87	0,75		*ALAS1* vs *GAPDH*	-6,84	1,10	
TBP vs *ALAS1*	1,47	0,64	**0,77**	*ALAS1* vs *RPS18*	-7,40	1,17	**1,15**
TBP vs *ACTB*	8,06	1,67		*ALAS1* vs *ACTB*	6,59	2,25	
TBP vs *B2M*	9,05	0,67		*ALAS1* vs *B2M*	7,58	0,91	
TBP vs *UBC*	-1,04	0,74		*ALAS1* vs *UBC*	-2,51	1,32	
TBP vs *RPII*	2,85	0,82		*ALAS1* vs *RPII*	1,38	1,10	
SDHA vs *TBP*	4,40	0,36		*ACTB* vs *TBP*	8,06	1,67	
SDHA vs *HPRT1*	-3,58	0,40		*ACTB* vs *SDHA*	3,66	1,48	
SDHA vs *GAPDH*	3,91	0,44		*ACTB* vs *HPRT1*	7,24	1,32	
SDHA vs *RPS18*	4,47	0,53		*ACTB* vs *GAPDH*	-0,25	1,55	
SDHA vs *ALAS1*	-2,93	0,85	**0,66**	*ACTB* vs *RPS18*	-0,80	1,24	**1,56**
HA vs *ACTB*	3,66	1,49		*ACTB* vs *ALAS1*	6,59	2,25	
SDHA vs *B2M*	4,65	0,44		*ACTB* vs *B2M*	0,99	1,76	
SDHA vs *UBC*	-5,44	0,58		*ACTB* vs *UBC*	-9,10	1,32	
SDHA vs *RPII*	-1,55	0,88		*ACTB* vs *RPII*	-5,21	1,48	
HPRT1 vs *TBP*	0,82	0,57		*B2M* vs *TBP*	9,05	0,67	
HPRT1 vs *SDHA*	-3,57	0,40		*B2M* vs *SDHA*	4,65	0,44	
HPRT1 vs *GAPDH*	7,49	0,60		*B2M* vs *HPRT1*	8,23	0,67	
HPRT1 vs *RPS18*	8,04	0,24	**0,71**	*B2M* vs *GAPDH*	0,74	0,26	**0,84**
HPRT1 vs *ALAS1*	0,65	1,02		*B2M* vs *RPS18*	0,18	0,77	

HPRT1 vs *ACTB*	7,24	1,32		*B2M* vs *ALAS1*	7,58	0,91	
HPRT1 vs *B2M*	8,23	0,67		*B2M* vs *ACTB*	0,99	1,76	
HPRT1 vs *UBC*	-1,86	0,75		*B2M* vs *UBC*	-10,09	0,77	
HPRT1 vs *RPII*	2,03	0,83		*B2M* vs *RPII*	-6,20	1,29	
GAPDH vs *TBP*	8,31	0,71		*UBC* vs *TBP*	-1,04	0,74	
GAPDH vs *SDHA*	3,91	0,44		*UBC* vs *SDHA*	-5,44	0,58	
GAPDH vs *HPRT1*	7,49	0,60		*UBC* vs *HPRT1*	-1,86	0,75	
GAPDH vs RSP18	0,56	0,70		*UBC* vs *GAPDH*	-9,35	0,61	
GAPDH vs *ALAS1*	-6,84	1,10	**0,81**	*UBC* vs *RPS18*	-9,91	0,80	**0,90**
GAPDH vs *ACTB*	-0,25	1,55		*UBC* VS *ALAS1*	-2,51	1,32	
GAPDH vs *B2M*	0,74	0,26		*UBC* vs *ACTB*	-9,10	1,32	
GAPDH vs *UBC*	-9,35	0,61		*UBC* vs *B2M*	-10,09	0,77	
GAPDH vs *RPII*	-5,46	1,30		*UBC* vs *RPII*	3,89	1,20	
RPS18 vs *TBP*	8,87	0,75		*RPII* vs *TBP*	2,85	0,82	
RPS18 vs *SDHA*	4,47	0,53		*RPII* vs *SDHA*	-1,55	0,88	
RPS18 vs *HPRT1*	8,04	0,24		*RPII* vs *HPRT1*	2,03	0,83	
RPS18 vs *GAPDH*	0,56	0,70	**0,78**	*RPII* vs *GAPDH*	-5,46	1,30	**1,08**
RPS18 vs *ALAS1*	-7,40	1,17		*RPII* vs *RPS18*	-6,02	0,85	
RPS18 vs ACTIN	-0,80	1,24		*RPII* vs *ALAS1*	1,38	1,10	
RPS18 vs *B2M*	0,18	0,77		*RPII* vs *ACTB*	-5,21	1,48	
RPS18 vs *UBC*	-9,91	0,80		*RPII* vs *B2M*	-6,20	1,29	
RPS18 vs *RPII*	-6,02	0,85		*RPII* vs *UBC*	3,90	1,20	

Supplementary material 3: Microscopic observation of mineral nodules formation for dGSCs at D7, D14, D21 and Alizarin Red S staining at D21. Bar scale=100μm.

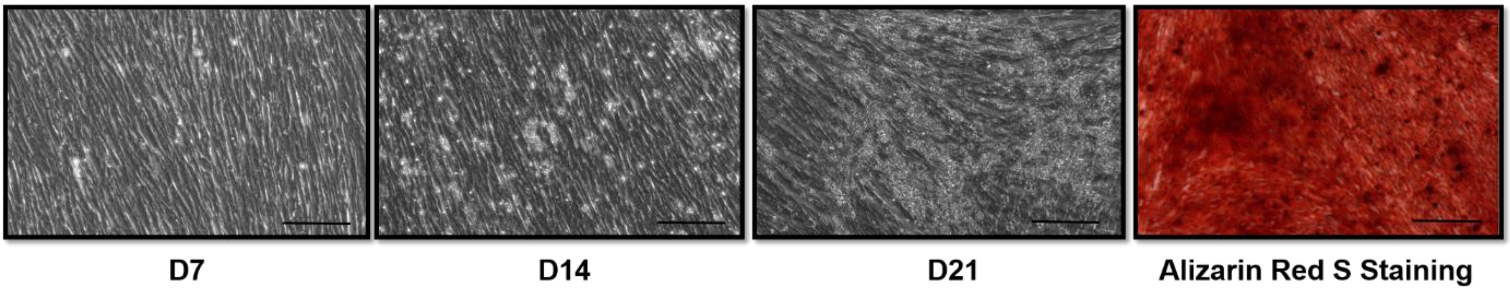

Supplementary material 4: MIQE checklist applied to our study

Item to check

ITEM		Yes/No	Details
Exprerimental design	Experimental and control groups	Yes	GSC dGSC ASC
	Number within each group	Yes	n=8 n=8 n=6
	Core or investigator's laboratory	Yes	Investigator's laboratory
	Acknowledgment of authors contributions	Yes	
Sample	description	Yes	
	Mass of sample processed	Yes	
	Processing procedure	Yes	
	Microdissection or macrodissection		
	If frozen how and how quickly	Yes	
	If fixed what and how quickly	Yes	
	Sample storage conditions and duration	No	
Nucleic acid extraction	Procedure and/or instrumentation	Yes	
	Name of kit	Yes	Promega®
	Source of additional reagents used	No	
	Details of DNAse RNAse treatment	No	
	Contamination assessment	Yes	
	Nucleic acid quantification	Yes	>100ng/µl
	Instrument and method	Yes	NanoDrop
	Purity	Yes	
	yield	Yes	
	RNA integrity	Yes	Agarose gel

Item to check

ITEM		Yes/No	Details
qPCR oligo-nucleotides	Primer sequences	Yes	
	RTPrimerDB identification number	No	
	Probe sequences	No	
	Location and identity of any modifications	No	
	Manufacturer of oligonucleotides	No	
	Purification method	No	
qPCR protocol	Complete reaction conditions	Yes	
	Reaction volume	Yes	15µl
	Amount of cDNA/DNA	Yes	15ng
	Primer concentration	Yes	250nM
	Polymerase identity and concentration	Yes	
	Buffer Kit manufacturer	No	
	Chemical composition of buffer	No	
	additives	Yes	Sybr GreenI
	Manufacturer of plates	Yes	Data not shown
	Thermocycling parameters	Yes	
	Reaction setup	Yes	Manual
	Manufacturer qPCR instrument	Yes	Bio-Rad®
qPCR validation	Evidence of optimization	No	
	Specificity	Yes	Melt
	Sybr Green I, C_q of NTC	Yes	Data not shown

			electrophoresis
	RIN/RQI or Cq	Yes	
	Electrophoresis traces	Yes	Figure 1
	Inhibition testing	Yes	
**Reverse transcription**	Complete reaction conditions	Yes	Superscript® II Reverse Transcriptase
	Amount of RNA	Yes	2µg
	Reaction volume	Yes	20µl
	Priming oligonucleotide	Yes	Random primers and oligo dT primers
	Reverse transcriptase	Yes	SuperScript enzyme
	Temperature and time	Yes	Manufacturer instruction
	manufacturer		(Invitrogen™)
	C_qs with and without reverse transcription	Yes	Data not shown
	Storage conditions of cDNA	Yes	Data not shown
**qPCR target information**	Gene symbol	Yes	Table 1
	Sequence accession number	Yes	Table 1
	Location of amplicon	No	
	Amplicon length	Yes	Table 1
	In silico specificity screen (blast, and so on)		
	Pseudogenes…	No	
	Sequence alignment	No	Data not shown
	Secondary structure analysis of amplicon	No	Data not shown
	Location of each primer by exon or intron	No	Data not shown
	Splice variants targeted	No	Data not shown

	Calibration curves	Yes	
	PCR efficiency	Yes	Table1
	CIs for PCR efficiency or SE	No	Data not shown
	I^2 of calibration curve	No	Data not shown
	Linear dynamic range	No	
	C_q variation at LOD	Yes	Table 1
	CIs throughout range	No	Data not shown
	Evidence of LOD	No	Data not shown
	If multiplex, efficiency and LOD of each assay	No	Data not shown
**Data Analysis**	qPCR analysis program	Yes	Bio-Rad®
	Method of C_q determination	Yes	Threshold value
	Outlier identification and disposition	Yes	Data not shown
	Results for NTCs	Yes	Data not shown
	Justification of number and choice of reference genes	Yes	4 algorithms
	Description of normalization methods	Yes	
	Number and concordance of biological replicates	Yes	
	Number and stage of technical replicates	Yes	
	Repeatability	Yes	
	Reproducibility	Yes	
	Power analysis	Yes	
	Statistical methods for results significance	Yes	
	Software , Cq or raw data submission with RDML	Yes	
